# The prognosis of non-small cell lung cancer patients according to endobronchial metastatic lesion

**DOI:** 10.1038/s41598-022-17918-1

**Published:** 2022-08-10

**Authors:** Yoonki Hong, Sunmin Park, Myoung Kyu Lee

**Affiliations:** 1 Department of Internal Medicine, School of Medicine, Kangwon National University Hospital, Kangwon National University, 1 Gangwondaehak-gil, Chuncheon, 24341 Republic of Korea; 2 Department of Internal Medicine, Yonsei University Wonju College of Medicine, 20, Ilsan-ro, Ilsan-dong, Gangwon-do Wonju-si, 26426 Republic of Korea; 3 Division of Pulmonary and Critical Care Medicine, Department of Medicine, Chuncheon Sacred Heart Hospital, Hallym University Medical Center, Chuncheon, Republic of Korea

**Keywords:** Medical research, Oncology

## Abstract

To evaluate the prognosis of non-small cell lung cancer (NSCLC) patients according to endobronchial metastatic lesion (EML), especially those not identified on positron emission tomography or computed tomography. We evaluated progression-free survival (PFS) and overall survival (OS) according to the presence of EML in patients with NSCLC who were diagnosed at a tertiary hospital between January 2010 and December 2019. A total of 364 patients were enrolled in this study. EML was found in 69 (19.0%) patients with NSCLC. In the patients with EML versus the patients without EML, median PFS was 7.0 (3.5–13.5) and 9.5 (5.5–17.5) months (*P* = 0.011), and median OS was 12.0 (6.0–30.0) versus 20.0 (10.0–39.0) months (*P* = 0.016), respectively. Median PFS and OS rates were highest in epidermal growth factor receptor (EGFR) (+) and EML (−) patients and lowest in EGFR (−) and EML (+) patients (*P* < 0.001). By multivariate cox regression analysis, PFS in overall patients with NSCLC was significantly associated with EML, EGFR mutation, performance status, and pleural effusion. NSCLC patients with EML had worse prognoses of PFS and OS than patients without EML.

## Introduction

Lung cancer is a leading health problem due to challenges in diagnosis and treatment, and, eventually results in poor prognosis^1^. Non-small cell lung cancer (NSCLC) is the most common histologic type, with a 5-year survival rate of all stages approximately 15%, although 5-year survival rates are higher when diagnosed at very early stages^1^. When we refer to lung cancer, early diagnosis with the least-invasive method and acquisition of sufficient tissue sampling are very important^2^.

Fibreoptic or flexible bronchoscopy plays an important role in the diagnosis and staging of lung cancer^3^. Bronchoscopy is mainly used as a diagnostic tool for tissue biopsy to determine the histological type and it also has an extended role in developing therapeutic modalities^4^.

Currently, positron emission tomography (PET)/computed tomography (CT) is known to be the most accurate diagnostic tool for lung cancer staging because it improves the detection of metastatic diseases, guides therapy, and predicts clinical outcomes^5,6^. However, several limitations must be considered when interpreting PET/CT findings, even though PET/CT has been shown to be a promising modality for lung cancer staging. In particular, artifacts in CT or PET images can be misdiagnosed if the lesion is adjacent to the diaphragm or heart. Such a discrepancy can cause the micro-lesion to not be observed and result in false negatives^7,8^.

An endobronchial metastatic lesion (EML) is considered present when infiltration of the bronchial epithelium by a malignant lesion is histologically consistent with the primary tumor. EML is an actually lesion that have metastasized to the central bronchi subsegmentally or more proximal to the extent of bronchoscopy^9^.

In particular, we have often diagnosed NSCLC with EML, especially those not identified on PET/CT. In this case, we had to modify the cancer stage according to the incidentally discovered EML that was histologically proven but not identified on PET/CT. However, there are few studies on EMLs not observed on PET/CT, and the treatment guidelines for these patients are still controversial.

Therefore, we performed a cohort study of patients diagnosed with NSCLC at our institution and investigated the characteristics of NSCLC patients with histologically proven EML whether there was a difference in disease-free survival (DFS), progression-free survival (PFS) and overall survival (OS) according to EML.

## Materials and methods

### Study design

We reviewed patients with NSCLC who were diagnosed at a tertiary hospital in Korea between January 1, 2010, and December 31, 2019. Eligible patients in this study were: (1) patients with histologically confirmed NSCLC; (2) age ≥ 19 years; and (3) those who underwent complete staging work-up including bronchoscopic and radiologic evaluation such as PET/CT.

Baseline demographics, disease characteristics (including age, sex, histologic type, stage, and Eastern Cooperative Oncology Group (ECOG) performance status (PS)), and clinical outcomes (including treatment and dates of diagnosis, surgery, and death or recurrence) were retrospectively obtained from medical records. Pretreatment clinical tumor-node-metastasis (TNM) classification was defined using the American Joint Committee on Cancer Staging Manual, eighth edition^10^. Smoking status was defined as follows: never (< 100 lifetime cigarettes) or current (quit < 1 year before diagnosis).

Exclusion criteria included: (1) previously diagnosed malignancy in addition to primary NSCLC, or previous or current illness thought likely to interfere with cancer treatment; (2) patients who did not complete the staging work-up including bronchoscopic and radiologic evaluation such as PET/CT; and (3) poor follow-up or follow-up performed in an outside institution. All patients gave their written informed consent for bronchoscopic evaluation, and Institutional Review Board of Yonsei University Wonju College of Medicine approved the analyses of the clinical and bronchoscopic data (IRB No. CR317051).

### Staging work-up and treatments

All registered patients underwent a pretreatment evaluation comprising physical examination, hematology, and biochemistry profiles, as well as obtaining patients’ complete historical information. Initial clinical staging was based on chest and brain CT, whole-body bone scans with single-photon emission, and abdominal CT or ultrasonography. We performed PET/CT to determine the TNM stage, and to screen for metastases that might not be identified by CT alone^11^.

We performed flexible bronchoscopy for all patients with suspected lung cancer. As shown by Rivera, flexible bronchoscopy became the recommended procedure for all patients suspected of having lung cancer^12^. Flexible bronchoscopy was done in a special procedure suite. Two expert pulmonologists (MK Lee, SM Park) performed flexible bronchoscopy using a BF-260 (Olympus, Tokyo, Japan) to confirm endobronchial cancerous lesions and reduce complications. We assessed the baseline epidermal growth factor receptor (EGFR) mutation status in adenocarcinoma patients by a direct sequencing of DNA extracted from samples of tumor tissue gathered during biopsy or resection, usually in the form of formalin-fixed paraffin-embedded diagnostic blocks^13^. The treatment strategy was established by the patient’s histology, molecular pathology, age, PS, comorbidities, and patient's preferences. Systemic therapies were performed in advanced-stage NSCLC patients with a PS of 0–2^14,15^.

### DFS, PFS and OS

We evaluated the characteristics of patients with NSCLC with histologically proven EML through bronchoscopy other than the primary cancer, especially those not identified on PET/CT, and evaluated DFS, PFS and OS according to the EML. We also compared PFS and OS according to EGFR status and EML in patients with lung cancer. Lastly, we investigated the prognostic factors associated with 1-year PFS and OS in patients with NSCLC according to EML. DFS was calculated from the length of time after primary treatment for a cancer ends that the patient survives without any signs or symptoms of that cancer. PFS was calculated from the start of the treatment until disease progression or death was confirmed. OS was defined as the duration from the date of diagnosis to the date of death or last follow-up.

### Statistical analysis

All statistical analyses were performed using SPSS for Windows software, ver. 26.0 (SPSS Inc., Chicago, IL). Descriptive statistics are expressed as median (interquartile range [IQR]) for continuous data and number (%) for categorical data. Wilcoxon rank-sum tests or t-tests were used to assess differences between NSCLC patients with and without EML, and between NSCLC patients with EML identified and not identified with PET/CT for continuous variables.

Pearson’s chi-square test or Fisher’s exact test was used to analyze categorical variables. Kaplan–Meier and log-rank tests were used to analyze DFS, PFS and OS between NSCLC patients with and without EML and between NSCLC patients with EML identified and not identified with PET/CT. Hazard ratios (HRs) and confidence intervals (CIs) were calculated using the Cox proportional hazard model. Univariate and multivariate analyses were performed to determine the prognostic factors associated with the 1-year PFS and OS in patients with NSCLC. Two-sided statistical tests were used, and *P*-values < 0.05 were defined as statistically significant.

### Ethics approval

This study was approved by the institutional review board of Yonsei University Wonju Severance Christian hospital (IRB No. CR317051).

### Consent to participate

Informed consent to participate in the study was obtained from all participants.

## Results

### Total subjects

**Figure 1:**
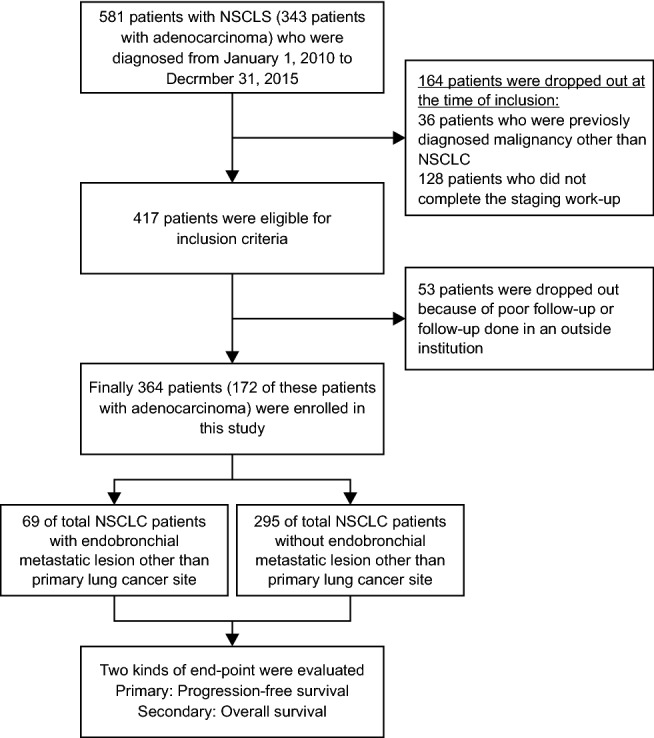
Flowchart for enrolled patients Flowchart illustrates selection process for patients with NSCLC who were diagnosed during the study period. *NSCLC* non-small cell lung cancer, *PET/CT* positron emission tomography/computed tomography.

Of the 581 patients with NSCLC (343 patients with adenocarcinoma) diagnosed during the study period, 36 patients who were previously diagnosed with malignancy in addition to NSCLC and 128 patients (41 with incomplete staging work-up and 87 who visited another hospital after diagnosis) who did not complete staging work-up including bronchoscopy and radiologic evaluations such as PET/CT, were excluded. 53 patients dropped out because they were not satisfied with the follow-up. Therefore, 364 patients (172 with adenocarcinoma) were enrolled in this study (Fig. 1). The median (IQR) age was 70 (63–75) years, 285 patients (78.3%) were male, and 299 (82.1%) were current or former smokers. The histologic subtypes were adenocarcinoma in 172 (47.3%), squamous cell carcinoma in 171 (47.0%), large cell carcinoma in 7 (1.9%), and others in 14 (3.8%) patients. The clinical characteristics of the patients are shown in Table 1.

**Table 1:** Characteristics of NSCLC patients according to EML.

Characteristics, N (%)	Patients with EML (N = 69)	Patients without EML (N = 295)	*P*-value
Age (year), median (IQR)	66 (61–75)	71 (63.5–76)	0.112
< 65	27 (39.1)	85 (28.8)	0.095
≥ 65	42 (60.9)	210 (71.2)	
**Gender**			
Male	54 (78.3)	231 (78.3)	0.994
Female	15 (21.7)	64 (21.7)	
**Smoking status**			
Current or former	58 (84.0)	241 (81.7)	0.652
Never	11 (16.0)	54 (18.3)	
**BMI, kg/m**^**2**^			
< 25.0	57 (82.6)	228 (77.3)	0.334
≥ 25.0	12 (17.4)	67 (22.7)	
**ECOG PS**			
0–1	35 (50.7)	183 (62.0)	0.084
≥ 2	34 (49.3)	112 (38.0)	
**Histologic subtype**			
SqCC	38 (55.1)	133 (45.1)	0.135
non-SqCC	31 (44.9)	162 (54.9)	
Adenocarcinoma	27 (39.1)	145 (49.2)	
Large cell carcinoma	0	7 (2.3)	
Others^a^	4 (5.8)	10 (3.4)	
**T stage**			
Tis—1	1 (1.4)	63 (21.4)	< 0.001
T2	7 (10.2)	111 (37.6)	
T3	5 (7.2)	43 (14.6)	
T4	56 (81.2)	78 (26.4)	
**N stage**			
N0	25 (36.2)	142 (48.1)	0.048
N1	7 (10.2)	27 (9.2)	
N2	18 (26.1)	59 (20.0)	
N3	19 (27.5)	67 (22.7)	
**M stage**			
M0	22 (31.9)	177 (60.0)	< 0.001
M1a	23 (33.3)	44 (14.9)	
M1b	11 (15.9)	30 (10.2)	
M1c	13 (18.9)	44 (14.9)	
**Clinical stage at baseline**			
0–IA	0	43 (14.6)	< 0.001
IB	4 (5.8)	41 (13.9)	
IIA	1 (1.4)	7 (2.4)	
IIB	1 (1.4)	24 (8.1)	
IIIA	5 (7.2)	35 (11.9)	
IIIB	3 (4.3)	10 (3.4)	
IIIC	6 (8.7)	17 (5.8)	
IVA	37 (53.6)	75 (25.4)	
IVB	12 (17.4)	43 (14.6)	
**Pleural effusion**			
Yes	15 (21.7)	65 (22.0)	0.958
No	54 (78.3)	230 (78.0)	
**Lung-to-lung metastasis**			
Yes	24 (34.8)	19 (6.4)	< 0.001
No	45 (65.2)	276 (93.6)	
**EGFR mutation**			
Exon 19 deletion	4 (5.8)	34 (11.5)	0.177
Exon 21 L858R	2 (2.9)	7 (2.3)	
Others^b^	0	3 (1.0)	
**Chemotherapy**			
Yes	53 (76.8)	190 (64.4)	0.049
No	16 (23.2)	105 (35.6)	
**Radiation therapy**			
Yes	31 (44.9)	113 (38.3)	0.311
No	38 (55.1)	182 (61.7)	
**Underlying disease**			
COPD	32 (46.4)	109 (36.9)	0.148
Hypertension	25 (36.2)	83 (28.1)	0.185
Diabetes mellitus	16 (23.2)	49 (16.6)	0.199
Tuberculosis	4 (5.8)	31 (10.5)	0.232
Angina	3 (4.3)	24 (8.1)	0.234

*BMI* body mass index,* COPD* chronic obstructive pulmonary disease, *ECOG PS* Eastern Cooperative Oncology Group performance status, *EGFR* epidermal growth factor receptor, *EML* endobronchial metastatic lesion, *IQR* interquartile range, *NSCLC* non-small cell lung cancer, *SqCC* squamous cell carcinoma, *y* age.

^a^11 (3 vs. 8) patients with non-small cell carcinoma, 2 (1 vs. 1) patients with pleomorphic carcinoma, and 1 (0 vs. 1) patient with spindle cell carcinoma.

^b^2 patients with the G719 mutation in exon 18 and one patient with the S768I mutation in exon 20.

### Characteristics of NSCLC patients with and without EML

EML was found in 69 (19.0%) of the total NSCLC patients, but was not found in 295 (81.0%) patients. The histologic subtypes were as follows: 27 (39.1%) adenocarcinoma patients with EML versus (vs) 145 (49.2%) patients without EML, squamous cell carcinoma 38 (55.1%) versus 133 (45.1%); large cell carcinoma, 0 (0%) versus 7 (2.3%); and others, 4 (5.8%) versus 10 (3.4%). EGFR mutations were observed in 6 (8.7%) patients with EML and 44 (14.9%) patients without EML (Table 1).

**Figure 2:**
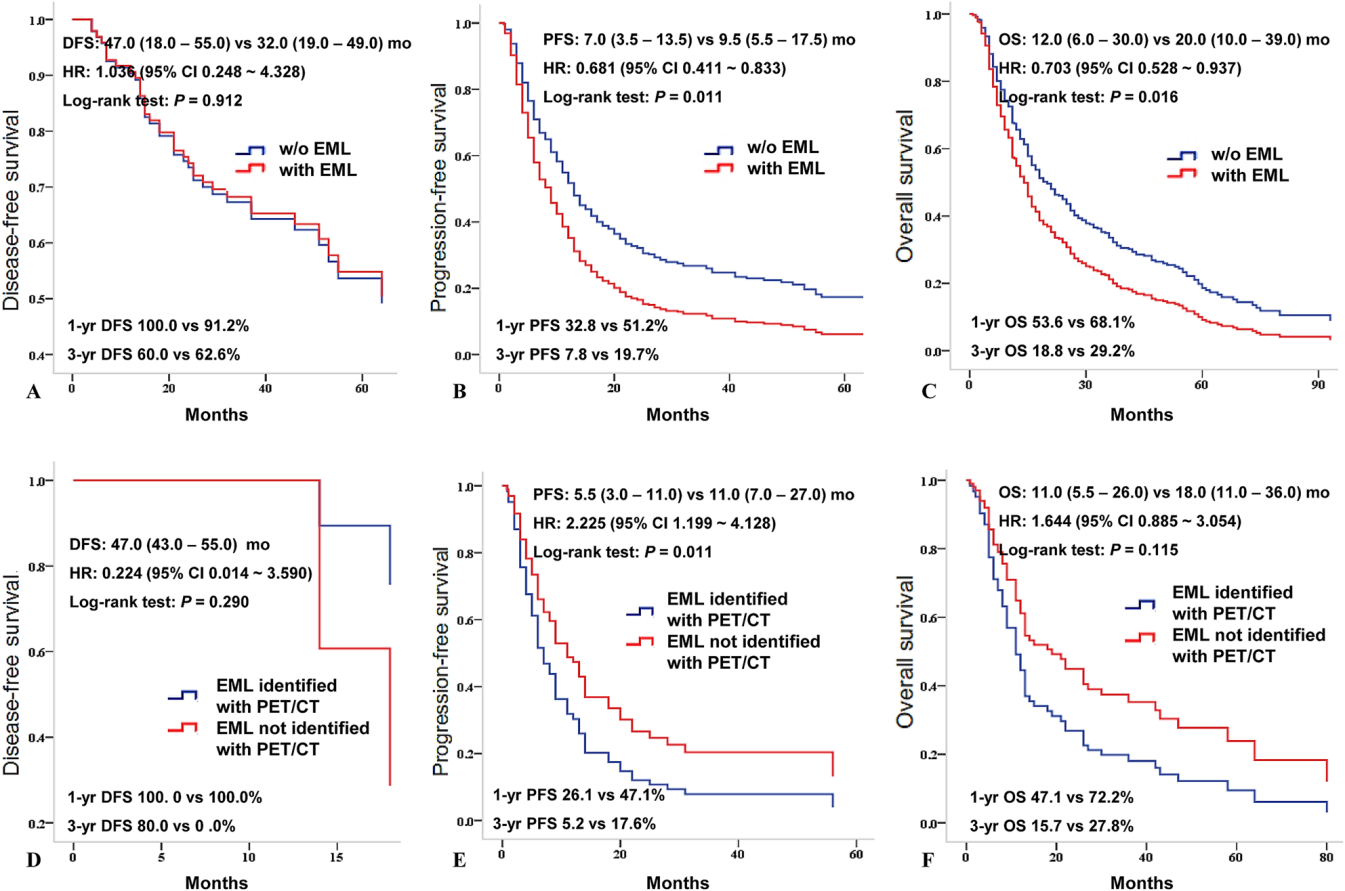
Median DFS, PFS and OS between NSCLC patients with and without EML other than primary cancer (N = 364) Median DFS was 47.0 (18.0–55.0) and 32.0 (19.0–49.0) months and in patients with EML compared to patients without EML (HR = 1.036, 95% CI 0.248–4.328, *P* = 0.912, (**A**); Median PFS (7.0 (3.5–13.5) vs. 9.5 (5.5–17.4) mo, HR = 0.681, 95% CI 0.411–0.833, *P* = 0.011, (**B)** and OS (12.0 (6.0–30.0) vs. 20.0 (10.0–39.0) mo, HR = 0.703, 95% CI 0.528–0.937, *P* = 0.016, (**C)** were significantly lower in patients with EML other than primary cancer. Median DFS (**D**), PFS (**E**) and OS (**F**) between NSCLC patients who have EML identified and not identified on PET/CT (N = 69). Median DFS 47.0 (43.0–55.0) mo vs. not checkable (HR = 0.224, 95% CI 0.014–3.590, *P* = 0.290, (**D**); Median PFS (5.5 (3.0–11.0) vs. 11.0 (7.0–27.0) mo, HR = 2.225, 95% CI 1.199–4.128, *P* = 0.011, (**E**); and OS 11.0 (5.5–26.0) vs. 18.0 (11.0–36.0) mo, HR = 1.644, 95% CI 0.885–3.054, *P* = 0.115, (**F**) between two groups. PFS was significantly higher in patients with EML not identified with PET/CT. *DFS* disease-free survival, *EML* endobronchial metastatic lesion, *HR* hazard ratio, *mo* months, *NSCLC* non-small cell lung cancer, *OS* overall survival rate, *PFS* progression-free survival rate, *vs.* versus.

Median DFS was 47.0 (18.0–55.0) and 32.0 (19.0–49.0) months and in patients with EML compared to patients without EML (HR = 1.036, 95% CI 0.248–4.328, *P* = 0.912) (Fig. 2A); Median PFS was 7.0 (3.5–13.5) and 9.5 (5.5–17.5) months and in patients with EML compared to patients without EML (HR = 0.681, 95% CI 0.411–0.833, *P* = 0.011), and median OS were 12.0 (6.0–30.0) and 20.0 (10.0–39.0) months (HR = 0.703, 95% CI 0.528–0.937, *P* = 0.016), respectively. Median PFS and OS were significantly lower in patients with EML than in patients without EML (Fig. 2B, C); when comparing patients with EML identified and not identified with PET/CT, DFS and OS were not significantly different as median DFS was 47.0 (43.0–55.0) months versus not checkable (HR = 0.224, 95% CI 0.014–3.590, *P* = 0.290) and median OS was 18.0 (11.0–36.0) versus 11.0 (5.5–26.0) months (HR = 1.644, 95% CI 0.885–3.054, *P* = 0.115), respectively (Fig. 2D, F). But, PFS was significantly higher in patients with EML not identified with PET/CT as median PFS was 5.5 (3.0–11.0) versus 11.0 (7.0–27.0) months (HR = 2.225, 95% CI 1.199–4.128, *P* = 0.011) (Fig. 2E).

**Figure 3:**
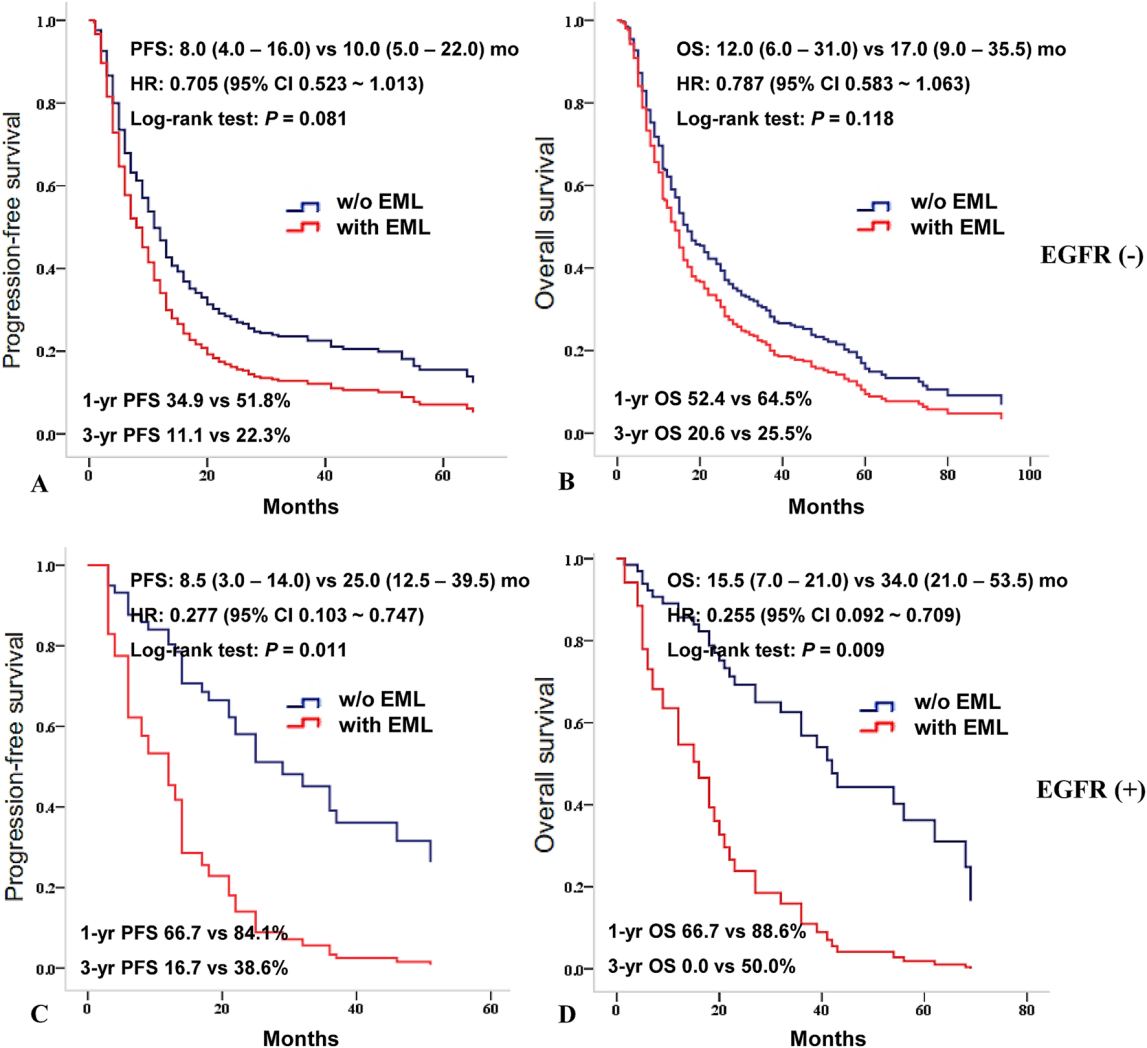
Median PFS and OS according to EML when EGFR negative or positiveWhen compared PFS and OS according to EML in case of EGFR negative, PFS (**A**) and OS (**B**) were not significantly different as median PFS was 8.0 (4.0–16.0) versus 10.0 (5.0–22.0) months (HR = 0.705, 95% CI 0.523–1.013, *P* = 0.081, (**A**), and median OS was 12.0 (6.0–31.0) versus 17.0 (9.0–35.5) months (HR = 0.787, 95% CI 0.583–1.063, *P* = 0.118, (**B**). When EGFR was positive, PFS (**C**) and OS (**D**) were significantly lower in patients with EML compared to patients without EML as median PFS was 8.5 (3.0–14.0) versus 25.0 (12.5–39.5) months (HR = 0.277, 95% CI 0.103–0.747, *P* = 0.011, (**C**), and median OS was 15.5 (7.0–21.0) versus 34.0 (21.0–53.5) months (HR = 0.255, 95% CI 0.092–0.709, *P* = 0.009, (**D**).*EGFR* epidermal growth factor receptor, *EML* endobronchial metastatic lesion, *HR* hazard ratio, *mo* months, *OS* overall survival rate, *PFS* progression-free survival rate, *vs.* versus.

When we compared PFS and OS according to EML in EGFR-negative patients, PFS and OS were not significantly different (Fig. 3A, B); However, when EGFR was positive, PFS and OS were significantly lower in patients with EML compared to patients without EML as median PFS was 8.5 (3.0–14.0) versus 25.0 (12.5–39.5) months (HR = 0.277, 95% CI 0.103–0.747, *P* = 0.011), and median OS was 15.5 (7.0–21.0) versus 34.0 (21.0–53.5) months (HR = 0.255, 95% CI 0.092–0.709, *P* = 0.009), respectively (Fig. 3C,D).

### Comparison between EML identified and not identified with PET/CT

Among the 69 patients with EML, 51 (73.9%) were identified with PET/CT, but 18 (26.1%) were not. When the characteristics of the patients were compared, there were no significant differences in age, sex, smoking status, and body mass index (BMI) between the two groups. However, ECOG showed better performance (*P* = 0.008), and the ratio of squamous cell carcinoma was higher in patients with EML not identified with PET/CT (*P* = 0.024) (Table 2). The characteristics of patients who had EMLs not identified with PET/CT are summarized (Supplementary Table S1). Supplementary Figure S1 shows an NSCLC patient who had EML not identified with PET/CT in the right lower lobe bronchus with a primary cancer site in the left upper lobe.

**Table 2:** Comparison between EML identified and not identified with PET/CT (N = 69).

Characteristics	EML not identified with PET/CT (N = 18)	EML identified with PET/CT (N = 51)	*P*-value
**Age (year)**			
< 65	10 (55.6)	17 (33.3)	0.097
≥ 65	8 (44.4)	34 (66.7)	
**Gender**			
Male	16 (88.9)	38 (74.5)	0.321
Female	2 (11.1)	13 (25.5)	
**Smoking status**			
Yes (Current or Former)	17 (94.4)	41 (80.4)	0.266
No	1 (5.6)	10 (19.6)	
**BMI, kg/m**^**2**^			
< 25.0	15 (83.3)	42 (82.4)	0.925
≥ 25.0	3 (16.7)	9 (17.6)	
**ECOG PS**			
0–1	14 (77.8)	21 (41.2)	0.008
≥ 2	4 (22.2)	30 (58.8)	
**Histologic subtype**			
SqCC	14 (77.8)	24 (47.1)	0.024
Non-SqCC	4 (22.2)	27 (52.9)	
**Clinical stage at baseline**			
0–IA	0	0	0.046
IB	1 (5.6)	3 (5.9)	
II	0	2 (3.9)	
IIIA	6 (33.3)	1 (2.0)	
IIIB	2 (11.1)	7 (13.7)	
IVA	9 (50.0)	26 (51.0)	
IVB	0	12 (23.5)	
**Endobronchial metastatic lesion**^**a**^			
Same lung	9 (50.0)	34 (66.7)	0.210
Different lung	9 (50.0)	17 (33.3)	
**Pleural effusion**			
Yes	0	15 (29.4)	0.007
No	18 (100.0)	36 (70.6)	
**EGFR mutation**			
Yes^b^	0	6 (11.8)	0.328
No	18 (100.0)	45 (88.2)	
**Chemotherapy**			
Yes	15 (83.3)	38 (74.5)	0.533
No	3 (16.7)	13 (25.5)	
**Radiation therapy**			
Yes	13 (72.2)	18 (35.3)	0.007
No	5 (27.8)	33 (64.7)	

*BMI* body mass index, *COPD* chronic obstructive pulmonary disease, *ECOG PS* Eastern Cooperative Oncology Group performance status, *EGFR* epidermal growth factor receptor, *EML* endobronchial metastatic lesion, *IQR* interquartile range, *NSCLC* non-small cell lung cancer, *SqCC* squamous cell carcinoma, *y* age.

^a^The site of primary cancer and endobronchial metastatic lesion.

^b^4 patients with Exon 19 deletion and 2 patients with L858R mutation in exon 21.

### Comparison according to EGFR and EML

**Figure 4:**
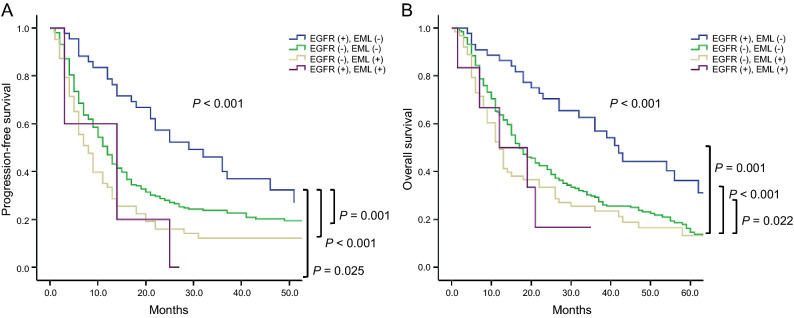
Median PFS according to EGFR and EML EGFR (−), EML (−) 11.0 (5.0–24.5); EGFR (+), EML (−) 25.0 (12.5–39.5); EGFR (−), EML (+) 8.0 (4.0–16.0); EGFR (+), EML (+) 8.5 (3.0–14.0) mo, respectively (*P* < 0.001). Median OS was also as follows; EGFR (−), EML (−) 17.0 (9.0–35.5); EGFR (+), EML (−) 34.0 (21.0–53.5); EGFR (−), EML (+) 12.0 (6.0–31.0); EGFR (+), EML (+) 15.5 (7.0–21.0) mo, respectively (*P* < 0.001). Both median PFS and OS were highest in EGFR (+) and EML (−). EGFR, epidermal growth factor receptor; *EML* endobronchial metastatic lesion, *mo* months, *OS* overall survival, *PFS* progression-free survival.

We compared PFS and OS according to EGFR and EML in patients with lung cancer. Median PFS was as follows; EGFR (−), EML (−) 11.0 (5.0–24.5); EGFR (+), EML (−) 25.0 (12.5–39.5); EGFR (−), EML (+) 8.0 (4.0–16.0); EGFR (+), EML (+) 8.5 (3.0–14.0) months, (*P* < 0.001). Median OS was also as follows; EGFR (−), EML (−) 17.0 (9.0–35.5); EGFR (+), EML (−) 34.0 (21.0–53.5); EGFR (−), EML (+) 12.0 (6.0–31.0); EGFR (+), EML (+) 15.5 (7.0–21.0) months, (*P* < 0.001). Both median PFS and OS were highest in the EGFR (+) and EML (−) groups. (Fig. 4A, B).

### Prognostic factors associated with PFS and OS in overall patients with NSCLC

We performed multivariate Cox regression analysis including parameters found to have *P*-values < 0.05 in univariate analysis. ECOG PS (HR 0.164, 95% CI 0.098–0.275, *P* < 0.001), EGFR mutation (HR 2.664, 95% CI 1.348–5.262, *P* = 0.005), pleural effusion (HR 0.497, 95% CI 0.356–0.695, *P* < 0.001) and EML (HR 0.630, 95% CI 0.441–0.899, *P* = 0.011) were associated with 1-year PFS. One-year OS was associated with ECOG PS (HR 0.181, 95% CI 0.119–0.276, *P* < 0.001), EGFR mutation (HR 2.171, 95% CI 1.006–4.684, *P* = 0.048), and pleural effusion (HR 0.634, 95% CI 0.430–0.935, *P* = 0.021) (Table 3). However, EML (HR 0.675, 95% CI 0.448–1.017, *P* = 0.060) was not associated with 1-year OS in multivariate analysis (Table 3).

**Table 3:** Prognostic factors associated with 1-year PFS and OS in NSCLC patients.

Variable	Univariate (1-year PFS)			Multivariate (1-year PFS )		
	95% CI	HR	*P*-value	95% CI	HR	*P*-value
Age, yr (< 65 vs0 ≥ 65)	0.447, 0.893	0.632	0.009*	0.504, 1.046	0.726	0.086
Gender (Female vs. Male)	0.938, 1.384	1.139	0.188			
Smoking status (Current or Former vs. Never)	0.896, 2.124	1.379	0.144			
ECOG PS (≤ 1 vs. ≥ 2)	0.146, 0.275	0.200	< 0.001*	**0.180, 0.348**	**0.250**	< 0.001*
Histologic subtype (SqCC vs. Non-SqCC)	0.890, 1.621	1.201	0.232			
BMI, kg/m^2^ (< 25.0 vs. ≥ 25.0)	1.083, 2.436	1.624	0.019*	0.755, 1.744	1.148	0.518
EGFR mutation	1.762, 6.749	3.448	< 0.001*	**1.348, 5.262**	**2.664**	0.005*
Pleural effusion	0.294, 0.557	0.405	< 0.001*	**0.356, 0.695**	**0.497**	< 0.001*
EML	0.419, 0.837	0.592	0.003*	**0.441, 0.899**	**0.630**	0.011*

Variable	Univariate (1-year OS)			Multivariate (1-year OS)		
	95% CI	HR	*P*-value	95% CI	HR	*P*-value
Age, yr (< 65 vs. ≥ 65)	0.387, 0.897	0.589	0.014*	0.530, 1.281	0.824	0.389
Gender (Female vs. Male)	0.897, 1.410	1.125	0.307			
Smoking status (Current or Former vs. Never)	0.804, 2.181	1.324	0.270			
ECOG PS (≤ 1 vs. ≥ 2)	0.101, 0.226	0.151	< 0.001*	0.119, 0.276	0.181	< 0.001*
Histologic subtype (SqCC vs. Non-SqCC)	0.738, 1.485	1.047	0.796			
BMI, kg/m^2^ (< 25.0 vs. ≥ 25.0)	1.043, 2.768	1.699	0.033*	0.738, 2.012	1.218	0.441
EGFR mutation	1.403, 6.447	3.008	0.005*	1.006, 4.684	2.171	0.048*
Pleural effusion	0.323, 0.680	0.469	< 0.001*	0.430, 0.935	0.634	0.021*
EML	0.411, 0.918	0.615	0.017*	0.448, 1.017	0.675	0.060

Significant values are in bold.

*BMI* body mass index, *ECOG PS* Eastern Cooperative Oncology Group performance status, *EGFR* epidermal growth factor receptor, *EML* endobronchial metastatic lesion, *HR* hazard ratio, *NSCLC* non-small cell lung cancer, *OS* overall survival rate, *PET/CT* positron emission tomography/computed tomography, *PFS* progression-free survival rate, *SqCC* squamous cell carcinoma, *vs*. versus, *yr* year.

*Indicates significance in univariate and multivariate analyses (*p* < 0.05).

## Discussion

This study shows that the survival rates in patients with NSCLC were significantly lower in patients with EML than in those without EML. This study is the first study to compare the prognosis between NSCLC patients with EML and NSCLC patients without EML confirmed using flexible bronchoscopy. This study suggests that it is important to confirm the presence of EML through flexible bronchoscopy during lung cancer evaluation in patients with NSCLC.

EML can cause airway complications, which can worsen cancer-related quality of life. Moreover, we found that it is associated with poor clinical outcomes. The frequency of endobronchial metastasis varies from 2 to 50% of pulmonary metastases from extra-thoracic malignancies^16,17^. Our study showed that the EGFR (+) and EML (−) patients had the highest median PFS and OS, whereas the EGFR (−) and EML (+) patients had the lowest (*P* < 0.001), as shown in Fig. 4. These results suggest that EML has an important effect on the PFS and OS of patients.

When EGFR was positive, PFS and OS were significantly lower in patients with EML compared to patients without EML whereas there was no significant difference in EGFR-negative patients. This means that EML can have a significant impact on prognosis, especially in EGFR-positive patients. Although it is not clear why EML affects the prognosis of the EGFR-positive group, EGFR mutation was associated with more frequent distant relapse and short PFS rate after neoadjuvant chemoradiation therapy followed by surgery in locally advanced N2-positive NSCLC^18,19^. In this study, lung-to-lung or bone metastasis was more prevalent in patients with EML, but not statistically significant as other variables.

When we compared ipsilateral EML to contralateral EML, PFS and OS were significantly higher in ipsilateral EML group. Obviously, the group with ipsilateral EMLs showed statistically lower bone, spine, and pleural metastasis than the contralateral group. Therefore, when contralateral EMLs are observed, there is a possibility that metastasis to other organs is accompanied.

Clinical presentations associated with EML include cough, shortness of breath, and hemoptysis^20^. Endobronchial metastases include (1) direct metastasis to the bronchus, (2) bronchial invasion by a parenchymal lesion, (3) mediastinal or hilar lymph node metastasis, and (4) the extension of a peripheral lesion along the proximal bronchus^21^. In the more advanced stages, the tumor tissue ulcerates through the epithelial layer, and the entire mucosal lining is gradually replaced by malignant tissue, resulting in bronchial stenosis^20^. Airway obstruction resulting in stenosis caused by endobronchial metastasis is a significant problem^22^, and as EML progresses, the chance of metastasis to other tissues becomes greater.

Although there was some disagreement between the CT findings and pathologic patterns of bronchial abnormality, a previous study demonstrated that chest CT helped detect superficial endobronchial lung cancer in 79% of cases^23^. However, there were no definite studies on how PET/CT could detect EMLs. In our study, we could identify that 73.9% of EMLs were identified using PET/CT.

Because of the increased reliance on PET/CT for cancer staging, it is vital that physicians be aware of pitfalls in PET/CT imaging to avoid misdiagnosis, over-staging, and unnecessary biopsies^24^. Although PET is useful in cancer staging, it showed a false-positive rate of 15–20% and false-negative rate of 9–28%^25^. In general, lesion size ≤ 2 cm and histologic subtype of lung cancer were significant factors associated with negative findings on PET^26^. Endobronchial lesions are mostly confined to superficial lesions and are small in size, which makes them unlikely to be detected on PET, and our study showed that 26.1% of total EMLs were not identified on PET/CT. When we compared the prognosis of NSCLC patients with and without EML other than the primary cancer, the median PFS and OS were significantly lower in patients with EML. Based on this result, it will be necessary to confirm EML in patients with lung cancer, especially in advanced stages. However, when we compared NSCLC patients with EML identified and not identified on PET/CT, the median OS showed no significant differences between the two groups although median PFS was significantly higher in patients with EML not confirmed by PET/CT.

Regarding prognostic factors associated with 1-year PFS and OS in patients with NSCLC; ECOG PS, EGFR mutation, pleural effusion, and EML were associated with 1-year PFS and OS, except for 1-year OS for EML. The ECOG PS is a strong independent prognostic factor for survival of patients with advanced NSCLC and remains the gold standard prognostic measure^27,28^ and our results also support this. For advanced NSCLC patients with EGFR mutations, especially exon 19 deletions, EGFR tyrosine kinase inhibitors were associated with better OS compared with conventional chemotherapy based on most studies including the phase III IPASS trial comparing gefitinib with doublet chemotherapy in the first-line setting^29,30^. Malignant pleural effusion is a common complication in patients with NSCLC and is associated with decreased survival in patients with distant metastases^31^.

In our study, EML with primary cancer, as well as ECOG PS and EGFR, were also significantly associated with poor prognosis in patients with NSCLC. Endobronchial metastasis originated from the primary cancer site via submucosal lymphatic or blood vessels rather than from carcinoma in situ^32^, which has been proposed to explain the development of multiple primary or locally recurrent cancers. In our study, many NSCLC patients with EML already showed advanced stages, such as lung-to-lung metastasis, which may have affected poor prognosis. This study is meaningful because it is the first to compare the prognosis of NSCLC patients with EML.

This study had some limitations. First, we included patients with EML confirmed by histological examination; therefore, we excluded patients who had EML but were not proven histologically. Second, PET uptake for EML may have been different according to the patient’s condition at the time of diagnosis, and it could be seen as a false negative; however, the PET/CT scan protocol was uniform, and patients were required to fast for at least 6 h prior to imaging. Third, this was a single-center retrospective study, and 217 of the total 581 patients were excluded because they had a previous history of treatment for cancer or did not complete the staging work-up. This may have affected the outcome of the study due to selection bias. In addition, treatment methods are diverse depending on clinicopathological characteristics, therefore, there will be limitations in interpreting PFS and OS because the stage or patient characteristics are different. Therefore, a multi-center and prospective study is needed to minimize bias.

## Conclusions

In this study, NSCLC patients with EML showed worse prognosis, including median PFS and OS, than those without EML. EML, ECOG PS, EGFR, and malignant pleural effusion were also significantly associated with poor prognosis. Therefore, it is important to confirm the presence of EML through flexible bronchoscopy during lung cancer evaluation in patients with NSCLC.

### Clinical practice points

Patients with non-small cell lung cancer (NSCLC) are sometimes diagnosed with endobronchial metastatic lesions (EMLs), especially those not identified on positron emission tomography/computed tomography. The cancer stage has to be modified according to the incidentally discovered histologically proven EML. This study shows that the PSF and OS in patients with NSCLC were significantly lower in patients with EML than in those without EML. This study is the first study comparing the prognosis between NSCLC patients with EML confirmed using flexible bronchoscopy and NSCLC patients without EML. This study suggests that it is important to confirm the presence of EML through flexible bronchoscopy during lung cancer evaluation in patients with NSCLC.

## Supplementary Information

Supplementary Information.

## Data Availability

The datasets used and/or analyzed during the current study are available from the corresponding author upon reasonable request. All methods were carried out in accordance with relevant guidelines and regulations.
