# Supplementary material for: The prognosis of non-small cell lung cancer patients according to endobronchial metastatic lesion
Source: Sci Rep. 2022 Aug 10;12:13588. doi: 10.1038/s41598-022-17918-1 (PMC9365769; doi:10.1038/s41598-022-17918-1)
Supplement: Supplementary file 1 — Supplementary Information. [file 41598_2022_17918_MOESM1_ESM.docx]

**Supplementary Materials**

**Supplementary Table S1**. NSCLC patients with EML not identified on PET/CT (N=18)

| Patient | Age (yr) | Gender | Histologic subtype | Histologic  grade | Primary cancer site on PET/CT | Endobronchial lesion not identified with PET/CT | ECOG PS | PET/CT  stage | Actual clinical stage | PFS (m) | OS (m) |
| --- | --- | --- | --- | --- | --- | --- | --- | --- | --- | --- | --- |
| 1 | 59 | F | Adenoca | Poor | LUL | RUL bronchus | 0 | T1bN0M0 | T1bN0M1a | 29 | 49 |
| 2 | 59 | M | SqCC | Moderate | LUL | RUL bronchus | 1 | T3N3M0 | T3N3M1a | 6 | 9 |
| 3 | 77 | M | SqCC | Poor | RLL | RML bronchus | 1 | T3N2M0 | T4N2M0 | 9 | 13 |
| 4 | 81 | M | SqCC | Moderate | LUL | RLL bronchus | 3 | T2aN2M0 | T2aN2M1a | 3 | 4 |
| 5 | 75 | M | SqCC | Poor | Rt. main | Trachea | 2 | T3N0M0 | T4N0M1a | 9 | 13 |
| 6 | 73 | M | SqCC | Poor | LUL | RUL bronchus | 3 | T2bN1M0 | T2bN1M1a | 3 | 6 |
| 7 | 62 | M | SqCC | Moderate | non-visible | Lt.main bronchus | 1 | TxN1M0 | T3N1M0 | 28 | 42 |
| 8 | 66 | M | Adenoca | Micropapillary | LLL | LLL bronchus | 1 | T1aN0M0 | T2aN0M0 | 18 | 40 |
| 9 | 75 | F | SqCC | Moderate | LLL | Trachea | 1 | T2aN0M0 | T4N0M0 | 13 | 22 |
| 10 | 57 | M | SqCC | Poor | RUL | LLL bronchus | 1 | T4N1M0 | T4N1M1a | 31 | 64 |
| 11 | 72 | M | SqCC | Poor | RLL | Trachea, RML bronchus | 2 | T3N0M0 | T4N0M0 | 8 | 14 |
| 12 | 58 | M | SqCC | Poor | RUL | LUL bronchus | 1 | T4N2M0 | T4N2M1a | 14 | 26 |
| 13 | 62 | M | SqCC | Well | RUL | LUL bronchus | 1 | T4N2M0 | T4N2M1a | 6 | 8 |
| 14 | 64 | M | NSCLC | Poor | RLL | RML bronchus | 0 | T1bN0M0 | T4N0M0 | 36 | 36 |
| 15 | 62 | M | SqCC | Moderate | RLL | RML bronchus | 1 | T2bN1M0 | T4N1M0 | 22 | 32 |
| 16 | 55 | M | Adenoca | Solid | RUL | RML bronchus | 0 | T2aN1M0 | T4N1M0 | 11 | 13 |
| 17 | 49 | M | SqCC | Poor | LUL | LLL bronchus | 0 | T3N2M0 | T4N2M0 | 27 | 32 |
| 18 | 67 | M | SqCC | Poor | LUL | LLL, RUL bronchus | 1 | T3N1M0 | T4N1M1a | 7 | 11 |

ECOG PS, Eastern Cooperative Oncology Group performance status; EML, endobronchial metastatic lesion; F, female; M, male; NSCLC, non-small cell lung cancer; OS, overall survival rate; PET/CT, positron emission tomography/computed tomography; PFS, progression-free survival rate; SqCC, squamous cell carcinoma;

**
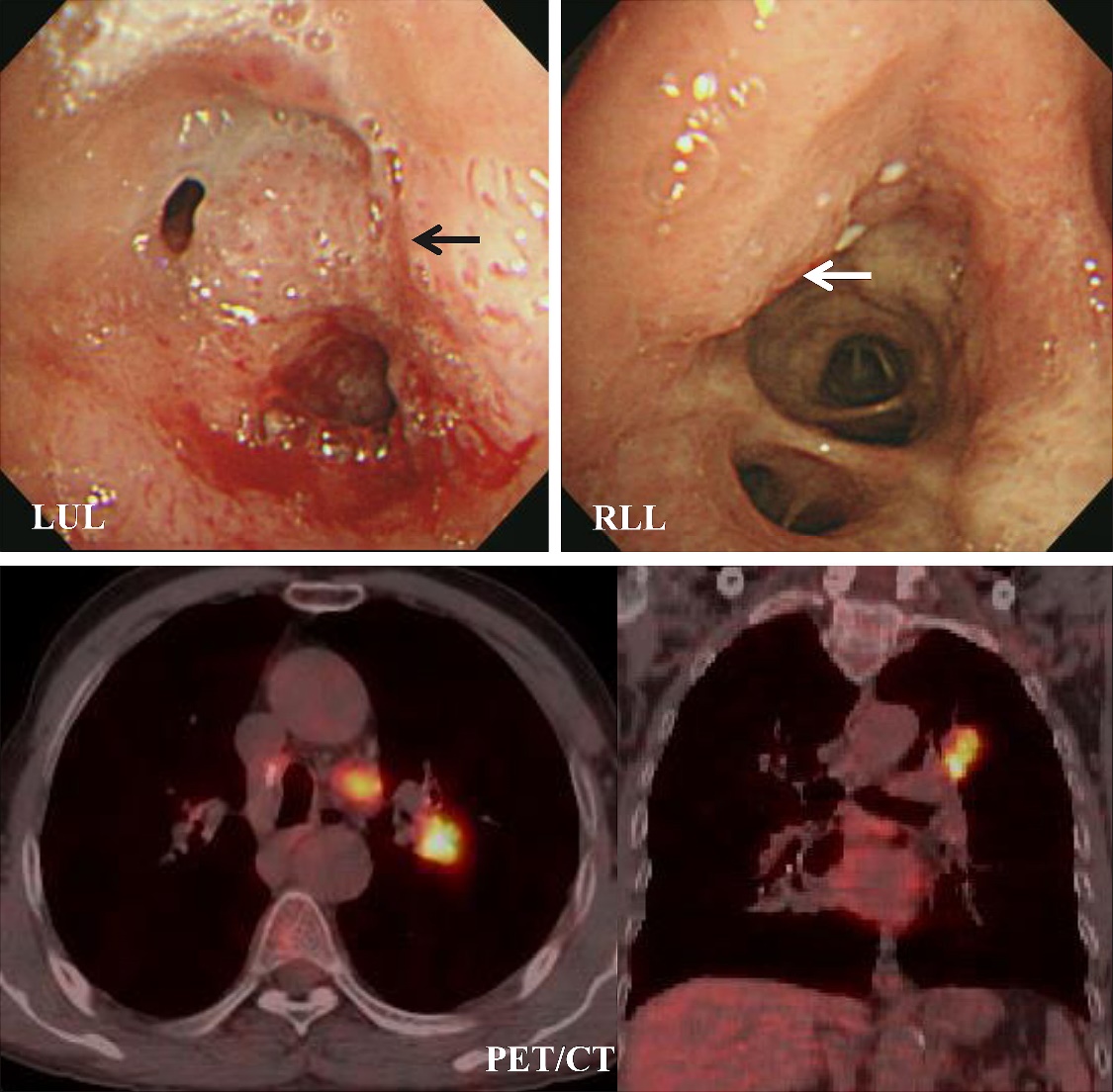
**

**Supplementary Figure S1**. A case of EML not identified on PET/CT in the RLL (white arrow) bronchus and primary lung cancer in the LUL (black arrow). EML, endobronchial metastatic lesion; LUL, left upper lobe; PET/CT, positron emission tomography/computed tomography; RLL, right lower lobe.
